# The relation of weight suppression and BMIz to bulimic symptoms in youth with bulimia nervosa

**DOI:** 10.1186/s40337-016-0111-5

**Published:** 2016-07-27

**Authors:** Erin C. Accurso, Jocelyn Lebow, Stuart B. Murray, Andrea E. Kass, Daniel Le Grange

**Affiliations:** 1 Department of Psychiatry, UCSF Weill Institute for Neurosciences, University of California, San Francisco, 3333 California Street, LH Suite 245, Box 0503, San Francisco, CA 94143 USA; 2 Department of Psychiatry and Behavioral Sciences, University of Miami Miller School of Medicine, Miami, FL USA; 3 Department of Psychiatry and Psychology, Mayo Clinic School of Medicine, Rochester, MN USA; 4 Department of Medicine, The University of Chicago, Chicago, IL USA; 5 Department of Pediatrics, University of California, San Francisco, CA USA

**Keywords:** Bulimia nervosa, Weight suppression, BMIz, Binge eating, Compensatory behaviors, Dietary restraint, Children, Adolescents

## Abstract

**Background:**

Weight suppression (WS), which is the difference between a patient’s highest and current weight, has been associated with bulimic symptom severity in adults with bulimia nervosa (BN). However, the impact of WS on eating disorder psychopathology in youth with BN is unknown.

**Methods:**

Participants included 85 youth with DSM-5 BN who presented for outpatient treatment. Current WS was calculated as the difference between highest and current body mass index z-score (BMIz), while greatest WS was the difference between highest and lowest BMIz, both assessed at participants’ current height. Separate multivariable linear regressions were conducted to determine if current or greatest WS was significantly associated with frequency of binge eating, compensatory behaviors, or dietary restraint. A secondary analysis was conducted on youth ages 16 and older, given the limitation of assessing WS at current height in younger participants with greater height instability.

**Results:**

Youth with higher levels of greatest WS (but not current WS) were older, had a longer duration of illness, and reported greater weight and shape concern. When adjusting for BMIz, neither current nor greatest WS was significantly associated with bulimic behaviors or dietary restraint in the full sample. However, in the subset of youth ages 16 and older, current WS moderated the effect of BMIz on binge eating and compensatory behaviors. For youth with high WS, those with a high current BMIz engaged in more frequent binge eating than those with low current BMIz, and the negative impact of BMIz on compensatory behaviors became weaker.

**Conclusions:**

Our findings suggest that WS is clinically relevant in the presentation of youth with BN, and that it may need to be addressed as one important factor in BN psychopathology. Future studies using growth charts to determine historically highest and lowest BMIz may help to further elucidate the link (or lack thereof) between WS and BN psychopathology in youth.

## Background

Weight suppression (WS)—the difference between a patient’s highest and current weight—may be an important factor in conceptualizing and treating bulimia nervosa (BN). Early theoretical models of BN in adults posit that dietary restraint contributes to binge eating and purging [1, 2]. Thus, WS resulting from restraint may exacerbate a psychobiological drive to engage in bulimic behavior. It is also likely that greater shape and weight concern and restraint predict greater WS, although research has been unable to examine these factors naturalistically as potential predictors of WS. Cross-sectional studies have found that WS is directly related to the frequency of binge eating and purging in treatment-seeking women with BN, even after adjusting for body mass index (BMI) and level of dietary restraint [3, 4]. High WS also predicted long-term increases in bulimic behavior in a community sample of women [5]. As a result, it has been suggested that WS may be a negative prognostic indicator for patients with BN. Indeed, high WS among adults with BN predicts treatment dropout [6, 7], longer time to remission [8], and the persistence of bulimic behaviors after treatment [6]. However, results are mixed, with several contrasting studies finding no relation between WS and treatment outcome [7, 9, 10].

BMI might moderate the WS-binge eating association, such that women with high WS and low BMI display the highest levels of binge eating [3]. This interaction may be the result of individuals with high WS and low BMI being more metabolically or biologically predisposed to engage in binge eating than those with high WS and a higher BMI, a profile which may instead increase attempts at dietary restraint and use of compensatory behaviors. Research also suggests that the effects of historical WS may be long-lasting, with greatest WS predicting binge eating frequency and its interaction with BMI predicting purging frequency [3] as well as predicting long-term weight gain [7].

Importantly, the link between WS and bulimic behaviors in the context of adolescent development is understudied. The impact of WS on the clinical presentation of youth may differ from that of adults because of an earlier age of symptom onset [11] and shorter duration of illness, with sustained WS potentially strengthening the relation between WS and BN psychopathology. Furthermore, operationalizing WS in youth is more complex since BMI change is expected with typical development. Because BMI percentile fails to adequately capture degree of weight change at the upper and lower percentiles, BMI z-score (BMIz) may be more appropriate to capture WS in youth.

The aims of the present study were 1) to describe the magnitude of WS in a treatment-seeking sample of youth with BN, 2) to explore the relation between WS (current and greatest) and other demographic and clinical factors, including eating disorder psychopathology, and 3) to examine the effect of WS on bulimic behaviors and dietary restraint, as well as the effect of WS x BMIz on bulimic behaviors and dietary restraint. We hypothesized that WS would be positively associated with bulimic behaviors, dietary restraint, and weight and shape concern, given that WS is likely driven by higher weight and shape concern and related dietary restraint, which is hypothesized as a main contributor to bulimic behaviors [1, 2]. Clarifying the relation between WS, bulimic behaviors, and dietary restraint may help to understand the model of BN psychopathology and inform treatment targets.

## Methods

Participants included 85 youth up to age 18 who were evaluated at The University of Chicago Eating Disorders Program between 1999 and 2014 and met DSM-5 criteria for BN [12]. Youth and at least one caregiver provided informed assent and consent, respectively.

### Assessment

#### Weight and height

Patients’ highest and lowest weights (with corresponding ages) at their current height were assessed by patient and caregiver self-report. All BMIz calculations were based on CDC norms for age and sex [13]. Current WS was calculated as the difference between highest BMIz and current BMIz, using weight and height measured at the assessment. Greatest WS was the difference between highest BMIz and lowest BMIz.

#### Eating disorder examination (EDE, version 12.0)

The EDE was administered as a diagnostic instrument, and its global and subscale scores were used to assess eating disorder pathology in the past month, including dietary restraint (Cronbach’s alpha = .733) and weight and shape concern (Cronbach’s alpha = .899, calculated by combining items on weight concern and shape concern subscales into one subscale, with the overlapping item included only once in the combined subscale) [14]. The EDE has demonstrated good reliability and validity [15, 16]. However, eating concern was not examined due to poor internal consistency within this sample (Cronbach’s alpha = .599).

### Analyses

IBM SPSS Statistics 22 was used for all analyses. Pearson correlations were examined between WS (current and greatest), BMIz (current and lowest), age, duration of illness, past month objective binge episode frequency, past month compensatory behavior frequency (i.e., vomiting, laxative misuse, diuretic misuse, and driven exercise), dietary restraint and weight and shape concern. Separate multivariable linear regressions were conducted to determine if WS (current or greatest) or its interaction with BMIz (current or lowest, respectively) were significantly associated with frequency of objective binge eating, compensatory behaviors, and dietary restraint in the past month. Independent variables were centered. Since our assessment of WS was contingent on height stability, leading to possible underestimation of WS in younger youth with more frequent changes in growth, we also re-ran these models in the subset of youth ages 16 and older (*n* = 60).

Missing data were not associated with age, gender, race, ethnicity, history of previous psychotherapy, previous hospitalization, percent of expected body weight (% EBW), or global EDE score (*p*s > .05). However, missing compensatory behavior data were associated with longer duration of illness (39.40 v. 23.43 months, *t* = −2.241, *p* = .029). Therefore, duration of illness was included as a covariate in analyses. Multiple imputation was used to handle missing data, including dietary restraint (*n* = 9, 10.6 %), objective binge eating episodes (*n* = 10, 11.8 %), compensatory behavior (*n* = 10, 11.8 %), lowest/highest BMIz (lowest: *n* = 26, 30.6 %; highest: *n* = 25, 29.4 %), and duration of illness (*n* = 13, 15.3 %).

## Results

Youth were mostly non-Latino (*n* = 71, 85.5 %) White (*n* = 76, 91.6 %) girls (*n* = 82, 96.5 %) with a mean age of 16.6 (*SD* = 1.5; range [12, 17]). Mean duration of illness was 23.5 months (*SD* = 19.8), and mean global EDE score was 3.59 (*SD* = 1.31). BMI z-scores were as follows: current (*M* = 0.46, *SD* = 0.79, range: [−1.34, 2.39]), lowest (*M* = −0.31, *SD* = 1.19, range: [−5.28, 2.16]), and highest (*M* = 1.15, *SD* = 0.69, range: [−0.87, 2.53]). On average, current WS was 0.75 standard deviations (*SD* = 0.72, range: [0.00, 3.07]), which represented a mean difference of 9.4 kg. Average greatest WS was 1.51 standard deviations (*SD* = 1.01, range: [0.26, 5.35]), which represented a mean difference of 18.1 kg. Greatest WS was higher in youth with a history of previous outpatient psychotherapy compared to no prior outpatient psychotherapy treatment (1.67 v. 1.08; *t* = −2.060, *p* = .044). There were no other differences in WS by gender, race/ethnicity, or history of outpatient psychotherapy or hospitalization (*p*s > .05).

### Eating disorder psychopathology

Current and greatest WS were not significantly correlated (*r* = .302, *p* = .060). Current WS was negatively correlated with current BMIz (*r* = −.640, *p* < .00001); similarly, greatest WS was negatively correlated with lowest BMIz (*r* = −.817, *p* < .00001). Greatest WS (but not current WS) was positively associated with age (*r* = .311, *p* = .007), duration of illness (*r* = .423, *p* = .0003), and weight and shape concern (*r* = .221, *p* = .048). Neither current nor greatest WS was correlated with dietary restraint or bulimic behaviors.

### Objective binge eating, compensatory behaviors, and dietary restraint

After adjusting for age, duration of illness, dietary restraint, and weight and shape concern, there were no significant effects of WS (current or greatest) or BMIz (current and lowest) on binge eating (*p*s > .05) in the full sample. However, in youth ages 16 and older (see Table 1a), there was a significant current BMIz x current WS interaction (*B* = 4.865, *SE* = 2.326, *t* = 2.091, *p* = .037) on binge eating, such that for older youth with high current WS, higher current BMIz was associated with more frequent binge eating than lower BMIz, but for those with low current WS, lower current BMIz was associated with more frequent binge eating than higher BMIz.

**Table 1:** Association between weight suppression (current and greatest) and objective binge eating, compensatory behaviors, and dietary restraint in the past month

1a. Objective Binge Eating	Current WS				Greatest WS			
	*B*	*SE*	*t*	*p*-value	*B*	*SE*	*t*	*p*-value
WS (current or greatest)	−0.18	2.32	−0.077	.94	0.41	2.61	0.155	.88
BMIz (current or lowest)	−1.10	2.49	−0.443	.66	−1.27	2.32	−0.546	.59
WS x BMIz	3.70	1.62	2.289	.023	0.67	1.48	0.451	.65
Age	−5.01	1.83	−2.742	.006	−5.29	1.85	−2.850	.005
Duration of illness	−0.05	0.08	−0.599	.55	−0.07	0.09	−0.779	.44
EDE shape and weight concern	4.38	1.30	3.374	.001	4.64	1.26	3.694	.001
EDE restraint	3.22	1.08	2.982	.003	3.19	1.09	2.933	.004
1b. Compensatory Behaviors	Current WS				Greatest WS			
	*B*	*SE*	*t*	*p*-value	*B*	*SE*	*t*	*p*-value
WS (current or greatest)	−0.60	3.34	−0.179	.86	−3.72	3.80	−0.979	.33
BMIz (current or lowest)	−8.99	3.58	−2.509	.013	−5.06	3.39	−1.491	.14
WS x BMIz	4.87	2.33	2.091	.037	−0.42	2.17	−0.194	.85
Age	1.09	2.63	0.415	.68	0.02	2.70	0.007	.99
Duration of illness	0.38	0.12	3.226	.001	0.43	0.12	3.470	.001
EDE shape and weight concern	11.91	1.87	6.378	.001	10.52	1.83	5.740	.001
EDE restraint	−1.18	1.56	−0.758	.45	−0.57	1.58	−0.360	.72
1c. Dietary Restraint	Current WS				Greatest WS			
	*B*	*SE*	*t*	*p*-value	*B*	*SE*	*t*	*p*-value
WS (current or greatest)	0.37	0.12	3.022	.003	0.34	0.14	2.468	.014
BMIz (current or lowest)	0.16	0.13	1.181	.24	0.30	0.12	2.422	.016
WS x BMIz	0.01	0.08	0.142	.89	0.06	0.08	0.734	.46
Age	0.05	0.10	0.471	.64	0.00	0.10	0.019	.99
Duration of illness	0.01	0.00	1.331	.18	0.01	0.01	1.290	.20
EDE shape and weight concern	0.72	0.06	12.900	.001	0.70	0.05	12.87	.001

After adjusting for age, duration of illness, dietary restraint, and weight and shape concern, there were no significant effects of WS (current or greatest) or BMIz (current and lowest) on compensatory behaviors (*p*s > .05) in the full sample. However, in youth ages 16 and older (see Table 1b), there was a significant main effect of current BMIz on compensatory behaviors (*B* = −8.987, *SE* = 3.581, *t* = −2.509, *p* = .013), such that older youth with a lower current BMIz engaged in more compensatory behaviors in the past month than those with a higher current BMIz. There was also a significant current BMIz x current WS interaction (*B* = 4.865, *SE* = 2.326, *t* = 2.091, *p* = .037), indicating that the effect of BMIz on compensatory behaviors was weaker for those with high current WS. There were no significant effects of greatest WS or lowest BMIz on compensatory behaviors (*p*s > .05).

After adjusting for age, duration of illness, and weight and shape concern, there was a significant main effect of current WS on dietary restraint (*B* = 0.374, *SE* = 0.124, *t* = 3.022, *p* = .003) in the full sample, such that youth with higher current WS reported higher levels of dietary restraint in the past month. In a separate parallel model, there were significant main effects of lowest BMIz (*B* = 0.229, *SE* = 0.093, *t* = 2.467, *p* = .014) and greatest WS (*B* = 0.277, *SE* = 0.114, *t* = 2.416, *p* = .016), such that both youth with higher lowest BMIz and higher greatest WS reported greater levels of dietary restraint in the past month. The pattern of results remained the same for both WS models in youth ages 16 and older (see Table 1c).

## Discussion

This study examined the association between WS and bulimic symptoms in a sample of treatment-seeking youth with BN. Youth with BN reported substantial WS at presentation to treatment (i.e., mean current WS was three-quarters of a standard deviation, representing 9.4 kg) as well as a history of substantial weight variability over time (i.e., mean greatest WS was one-and-a-half standard deviations, representing 18.1 kg), based on the CDC population BMI distribution [13]. Interestingly, current WS and greatest WS were highly negatively associated with current BMIz and lowest BMIz, respectively, despite non-significant associations in adults with BN [3, 5, 17, 18]. This may reflect the fact that youth with BN who have low current BMIz or low historical BMIz engage in weight loss behaviors developmentally earlier or more effectively than those with higher BMIz, resulting in greater current or historical weight loss, whereas those with higher BMIz may not yet have effectively engaged in behaviors that lead to WS, regardless of possible efforts to do so. In contrast, the non-significant association found in adults indicates that similar levels of WS are achieved across the BMI distribution, possibly related to greater age and more enduring attempts to suppress weight.

In keeping with WS findings in adults with BN, multivariable analyses with older youth suggest that as current WS increases, those with a high current BMIz engage in more frequent binge eating, while those with a low current BMIz engage in less frequent binge eating. This may highlight the fact that youth with lower BMIz and high WS have been more “successful” at restriction or that abstaining from binge eating becomes easier at a lower BMIz, potentially due to a dampening of hunger cues at a highly suppressed low weight. Conversely, youth with higher BMIz and high WS might be less “successful” at restricting, and more susceptible to binge eating. This suggests that WS may have a greater effect on youth with relatively higher premorbid BMIz.

Current WS was also related to compensatory behaviors in older youth with BN, in that lower current BMIz was associated with more frequent compensatory behaviors than higher current BMIz. Though the impact of BMIz on compensatory behaviors was weakened in youth with higher current WS, this finding likely suggests that youth who engage in more compensatory behaviors achieve lower BMIz, rather than the alternative potential explanation that youth with low BMIz engage in more compensatory behaviors. However, the impact of BMIz on compensatory behaviors was diminished by WS, which may reflect the fact that compensatory behaviors are less effective for maintenance of low weight in youth with higher WS.

Current and greatest WS were also associated with higher levels of dietary restraint, such that a history of WS predicts later dietary restraint, in addition to the contemporaneous relation between WS and restraint. This suggests that youth who were previously successful at restricting their intake (i.e., achieved high historical WS) are likely to continue engaging in higher levels of dietary restraint. In other words, historical WS can be viewed as a potential risk factor for ongoing dietary restraint. The relation between current WS and dietary restraint also suggests that youth with BN are relatively successful in their efforts to restrict. Despite small to medium correlations between greatest WS and older age, longer duration of illness, and greater weight and shape concern, greatest WS was not associated with bulimic behaviors after adjusting for these variables, despite the association found in adults with BN [3]. Together, these findings suggest that current WS is important in the conceptualization of BN psychopathology in youth, whereas historical WS (i.e., greatest WS) is less relevant given its association with dietary restraint only.

To our knowledge, this is the first study of WS in a sample of youth with BN. In keeping with the adult literature, analyses examined the potential interaction between WS and BMIz and adjusted for dietary restraint, as well as additional factors associated with WS (e.g., age, duration of illness, and weight and shape concern). Several limitations include the use of cross-sectional data within a modestly-sized treatment-seeking sample with missing data on weight history and the inability to examine WS in relation to eating concern. Furthermore, WS calculations were limited to using youth’s current height, which may have underestimated greatest WS. Reliance on self-report may have further biased WS estimates. Indeed, the discrepant results for youth ages 16 and older suggest that this method of assessing WS might have been inadequate for youth with more frequent changes in height (using age as a proxy for height stability). WS assessment in developing youth still growing in height may require a more sophisticated, developmentally sensitive approach given the need to account for age, height, and weight to properly calculate WS at times of interest. Rather than assessing highest and lowest weight, patient’s historical BMI-for-age growth charts should be used to determine highest and lowest BMI percentiles, using multiple weights, heights, and ages from medical records to calculate BMIz. Comprehensive growth charts for each patient would have provided a better estimate of WS, which was unfortunately not possible in the current study.

## Conclusions

This study suggests that WS is an important marker of BN psychopathology in youth, which should be assessed at the start of treatment. Given the challenges in assessing WS in developing youth, additional studies using more complete records of WS history (i.e., BMI-for-age growth charts) are needed to further clarify the impact of WS on the development and maintenance of bulimic symptoms in youth. Future studies should also examine its prognostic value in youth, given some research in adults suggesting that WS may be related to poorer treatment outcomes.

## Abbreviations

BMI, body mass index; BMIz, BMI z-score; BN, bulimia nervosa; DSM, diagnostic and statistical manual for mental disorders; EDE, eating disorder examination; M, mean; SD, standard deviation; WS, weight suppression
